# The characteristics and optimal treatment of urolithiasis associated with tuberous sclerosis complex

**DOI:** 10.1007/s11255-021-02871-1

**Published:** 2021-04-25

**Authors:** Takashi Hatano, Katsuhisa Endo

**Affiliations:** 1 Department of Urology, Seirei Yokohama Hospital, 215 Iwai-cho Hodogaya-ku, Yokohama, Kanagawa 240-8521 Japan; 2 Department of Urology, JR Tokyo General Hospital, Tokyo, Japan

**Keywords:** Urolithiasis, Tuberous sclerosis complex, Renal angiomyolipoma, Topiramate, Zonisamide, Urine specific gravity

## Abstract

**Purpose:**

The most common renal symptoms of tuberous sclerosis complex (TSC) are angiomyolipomas (AMLs) and renal cysts; however, some patients with TSC also develop urolithiasis. We retrospectively investigated the characteristics and treatment of urolithiasis associated with TSC.

**Methods:**

We analyzed 142 patients who met the diagnostic criteria for TSC, of whom 20 (14.1%) had urolithiasis. We compared the patients’ characteristics, urinary specific gravity, urine pH, serum calcium and intact parathyroid hormone in the urolithiasis and non-urolithiasis groups. In the urolithiasis group, the stone characteristics and various treatments were analyzed.

**Results:**

The antiepileptic drugs topiramate and zonisamide were more frequently administered to the urolithiasis group than the non-urolithiasis group (*P* = 0.013, *P* = 0.048, respectively). The urine specific gravity and urine pH levels were higher in the urolithiasis group than in the non-urolithiasis group (*P* = 0.005, *P* = 0.042, respectively). A multivariate logistic regression analysis demonstrated that urine-specific gravity (*P* = 0.018; odds ratio 1.471; 95% confidence interval 1.098–1.872) was a significant predictor of TSC-associated urolithiasis. Four patients could not receive extracorporeal shock wave lithotripsy due to the risk of bleeding from the AML.

**Conclusion:**

Patients with TSC who have an increased urine specific gravity, alkaline urine, and a longer administration of topiramate and zonisamide tend to demonstrate an increased risk of developing urolithiasis and therefore such cases require adequate care. If urolithiasis is comorbid with TSC-associated AML, the treatment options are more limited in cases with multiple AMLs around the stone due to an increased risk of hemorrhage.

## Introduction

Tuberous sclerosis complex (TSC) is an autosomal dominant genetic disorder in which hamartomas develop throughout the body, and is associated with various organ disorders [1]. TSC arises from a mutation of either the *TSC1* gene on chromosome 9 or the *TSC2* gene on chromosome 16, which encode hamartin and tuberin, respectively [2, 3]. Anomalies in the *TSC1* and *TSC2* genes, which are important regulators of the mammalian target of rapamycin pathway, results in the development of benign and/or malignant tumors in multiple organs [4]. In Japan, the incidence of TSC is estimated to be one in 7000–10,000 persons, and the total number of patients is estimated to be 15,000, which is equivalent to the frequency in the US [5]. The number of TSC patients without symptoms is increasing [6].

The most common renal manifestations of TSC are angiomyolipomas (AMLs), which are observed in up to 80% of the patients [7]. Renal cysts are another frequent kidney lesion in TSC, and are observed in 14–32% of the patients; however, the incidence of urolithiasis in TSC is unknown [8]. Since both urolithiasis and rupture of renal AML cause severe pain and hematuria, discrimination between them is very important for the management of TSC. However, there have been few reports on urolithiasis associated with TSC. Furthermore, carbonic anhydrase inhibitors such as topiramate and zonisamide are commonly used as a treatment for epilepsy, and these drugs can induce metabolic acidosis in some patients. It has been reported that long-standing metabolic acidosis promotes urolithiasis [9]. In the present study, we retrospectively investigated the characteristics and optimal treatment of urolithiasis associated with TSC.

## Methods

### Patients and study design

TSC was diagnosed according to the ITSCCG diagnostic criteria after consultations with an internist and a dermatologist. Between 2012 and 2019, of the 142 patients who met the diagnostic criteria, 20 had urolithiasis. One hundred twenty-two patients without urolithiasis were used as controls. Urolithiasis was diagnosed based on abdominal CT.

Urine specific gravity, urine pH, serum calcium, and intact parathyroid hormone were retrospectively analyzed in the urolithiasis and non-urolithiasis groups. A BioMajesty JCA-BM6070 biochemical analyzer (JEOL, Tokyo, Japan) was used for the analysis. When the serum albumin level was ≤ 4 mg/dL, the serum calcium levels were corrected using the formula: calcium + (4—“serum albumin”), and designated as corrected calcium. All test values ​​are shown as the average of three different measurements taken on different days.

In the urolithiasis group, the stone size, location, and various treatments were analyzed. The information on the treatment status of epileptic seizures and the use of antiepileptic agents of all patients was obtained by interviews. This study was approved by the ethics committee of JR Tokyo General Hospital. All patients gave informed consent.

### Exclusion criteria

The following patients were excluded: those with poor respiratory conditions as a result of lung lymphangioleiomyomatosis, those with swallowing disorders, pregnant patients, and those taking vitamin D3 or calcium.

### Statistical analyses

Patient characteristics and laboratory findings were assessed using Student’s *t*-test or the Wilcoxon signed-rank test. *P* values of < 0.05 were considered to indicate statistical significance. All statistical analyses were performed using the SPSS software program (version 23.0, SPSS, Chicago, IL, USA).

## Results

### Patient characteristics

We analyzed a total of 142 patients with TSC, 20 of whom (14.1%) had urolithiasis (Table 1). No significant differences in age, sex, body mass index or incidence of diabetes were found between the two groups. The incidence of mental retardation in the urolithiasis group was higher than in the non-urolithiasis group (*P* = 0.027). Epileptic seizures were observed in > 80% of cases in both groups, and the patients were treated with antiepileptic agents. Topiramate and zonisamide were more frequently administered in the urolithiasis group than in the non-urolithiasis group (*P* = 0.013, *P* = 0.048, respectively). Sixteen of the 20 cases in the urolithiasis group were treated with topiramate or zonisamide. No patients took both agents. The median periods of antiepileptic treatment were longer than 80 months in both groups. No significant differences were found between the two groups with regard to the administration rates of other antiepileptic agents, the treatment period, or the incidence of manifestations of TSC in the kidney, skin, brain, and other organs.

**Table 1:** Patient characteristics

Characteristics	Urolithiasis group (*n* = 20)	Non-urolithiasis group (*n* = 122)	*P* value
Median age (range)	27 (17–51)	26 (16–56)	0.609
Sex			
Male/female	10/10	63/59	1.000
Mean body mass index	24.7	24.0	0.424
Diabetes	2 (10%)	12 (10%)	1.000
Mental retardation	16 (80%)	63 (52%)	0.027
Epileptic seizure	18 (90%)	99 (81%)	0.366
Antiepileptic agents			
Carbamazepine	12 (60%)	50 (41%)	0.145
Zonisamide	9 (45%)	27 (22%)	0.048
Topiramate	7 (35%)	14 (11%)	0.013
Sodium valproate	7 (35%)	33 (27%)	0.592
Levetiracetam	6 (30%)	26 (21%)	0.395
Clobazam	4 (20%)	17 (14%)	0.499
Median treatment period with antiepileptic agents (months) (range)	102 (40–184)	84 (26–240)	0.245
Kidney			
Angoimyolipoma	17 (85%)	82 (67%)	0.124
cyst	6 (30%)	24 (20%)	0.374
Skin			
Facial angiofibromas	17 (85%)	96 (79%)	0.765
Hypomelanotic macules	9 (45%)	64 (52%)	0.632
Shagreen patch	6 (30%)	40 (33%)	1.000
Brain			
Cortical tubers	19 (95%)	100 (82%)	0.198
Subependymal nodules	13 (65%)	73 (60%)	0.806
Subependymal giant cell astrocytoma	3 (15%)	12 (10%)	0.445
Others			
Lung lymphangioleiomyomatosis	5 (25%)	36 (30%)	0.794
Retinal hamartoma	4 (20%)	14 (11%)	0.285

### Laboratory findings

Table 2 shows the urinary and serum findings in both groups. The urine specific gravity and urine pH levels were higher in the urolithiasis group than in the non-urolithiasis group (*P* = 0.005, *P* = 0.042, respectively). No significant differences were found between the serum corrected calcium and intact parathyroid hormone levels of the two groups. A multivariate logistic regression analysis showed that urine specific gravity (*P* = 0.018; odds ratio 1.471; 95% confidence interval 1.098–1.872) was a significant predictor of TSC-associated urolithiasis (Table 3).

**Table 2:** The laboratory findings in the urolithasis and non-urolithiasis groups

	Urolithiasis group	Non-urolithiasis group	*P* value
Urine specific gravity	1.026 ± 0.007	1.017 ± 0.005	0.005
Urine pH	7.4 ± 0.6	6.9 ± 0.5	0.042
Serum corrected calcium (mg/dl)	9.3 ± 0.5	9.7 ± 0.5	0.086
Serum intact PTH (pg/ml)	44.3 ± 19.5	40.8 ± 12.9	0.781

*PTH* parathyroid hormone

**Table 3:** The multivariate logistic regression analysis of risk factors for tuberous sclerosis complex-associated urolithiasis

	*P* value	Odds ratio (95% confidence interval)
Mental retardation	0.480	0.789 (0.509–1.524)
Zonisamide	0.514	1.024 (0.945–1.190)
Topiramate	0.107	1.789 (0.882–3.636)
Urine specific gravity	0.018	1.471 (1.098–1.872)
Urine pH	0.233	1.490 (0.774–2.905)

### Characteristics of the stones and treatments of the urolithiasis group

**Fig. 1:**
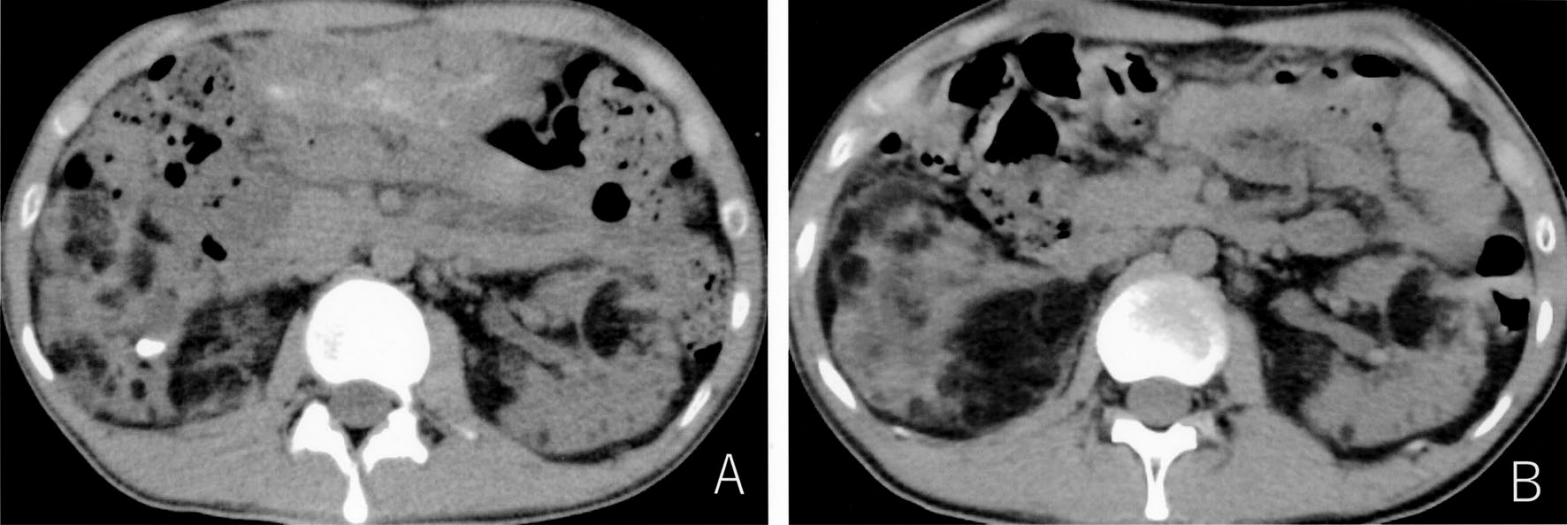
Axial CT of the kidney. **a** A 13 mm stone can be seen in the right kidney. Multiple AMLs are observed around the stone. **b** The stone was completely removed after transurethral lithotripsy

Table 4 shows the characteristics of the stones and treatments of the urolithiasis group. At the time of the diagnosis, 15 patients had symptoms and 5 had no symptoms. Four cases had bilateral urolithiasis. The median stone size was 8 mm; 11 were renal and 13 were ureteral stones. Nine cases (45%) had stones of ≤ 6 mm in diameter; no patients had stones ≥ 20 mm in diameter. The stone treatments included oral medication (*n* = 13), surgery (*n* = 5) and active surveillance (*n* = 7). Four of the 5 patients who underwent surgery had multiple AMLs around the stone. Because of the risk of bleeding from AML, these patients could not undergo extracorporeal shock wave lithotripsy (ESWL); thus, transurethral lithotripsy (TUL) was performed. All 5 patients who received surgery became stone-free. Six of the 13 cases treated with oral medication had spontaneous stone expulsion. The stone composition was pure calcium oxalate in only one case and mixed calcium phosphate and calcium oxalate in 10 cases. A typical case is shown in Fig. 1. The subject was a 25-year-old man. He had a subependymal giant cell astrocytoma in the brain, multiple AMLs in the bilateral kidneys, and multiple angiofibroma on the face. He visited our hospital in June 2017 due to macrohematuria. Abdominal plain CT showed a 13-mm stone in the right kidney. ESWL was difficult because AMLs surrounded the stone. Thus, TUL was performed and the stone was completely removed. All patients who discontinued receiving either topiramate or zonisamide showed an improvement in their urine alkaline levels. Their mean urine pH decreased from 7.6 ± 0.4 to 7.1 ± 0.2 and no recurrence of stones has been observed to date. In addition, no new stones were found in patients who drank > 2 L of water per day.

**Table 4:** The characteristics of the stones and treatments of the urolithiasis group

Symptoms	
Abdominal pain	7
Back pain	6
Macrohematuria	4
Microhematuria	3
No symptoms	5
Stone side	
Right/Left/Bilateral	9/7/4
Size	
≤ 3 mm	1
3.1–6 mm	8
6.1–10 mm	7
10.1–20 mm	4
> 20 mm	0
Location	
R2/R3	8/3
U1/U2/U3	6/2/5
Treatment	
Anticholinergic agents	9
Alpha 1 blocker	4
Transurethral lithotripsy	4
Extracorporeal shock wave lithotripsy	1
Active surveillance	7

*R2* stones in the renal pelvis and calix, *R3* stones in the ureteropelvic junction, *U1* stones in the upper ureter, *U2* stones in the middle ureter, *U3* stones in the distal ureter

## Discussion

The incidence of epileptic seizures was 80–90% in TSC patients [10, 11]. Refractory epileptic seizures are often observed in TSC, and it is not uncommon to take three or more antiepileptic agents [10, 11]. Topiramate and zonisamide have been widely used as antiepileptics. However, it has been reported that those drugs are associated with the development of acidosis and urolithiasis [12, 13]. In some patients, topiramate and zonisamide lead to renal tubular acidosis through the inhibition of carbonic anhydrase in the renal tubules, which influences systemic metabolic acidosis and alkaline urine pH with a low urine citrate concentration. These metabolic changes result in calcium phosphate stone formation [12, 13]. In our study, the urine pH levels were higher in the urolithiasis group than in the non-urolithiasis group. A study by Maalouf et al. showed that the prevalence of symptomatic urolithiasis among adult topiramate users was 10.7% [14]. The median daily dose of topiramate was 300 mg and the median treatment period was 48 months [14]. Faught reported that the incidence of kidney stones associated with zonisamide was 2.7% (15 of 549) [15]. Most patients with refractory epileptic seizures need to take antiepileptic agents for a long period. Longer administration of topiramate and zonisamide might result in a higher incidence of urolithiasis [12, 14, 15]. In our study, the median antiepileptic treatment period was > 80 months in both groups. We discontinued both drugs for all patients who had urolithiasis and switched to other agents.

In the urolithiasis group, the incidence of mental retardation and the urine specific gravity were higher than in the non-urolithiasis group. Patients with mental retardation are susceptible to dehydration because they cannot keep themselves hydrated. Thus, the daily urine volume decreased and the urine specific gravity increased. Increased urine specific gravity may promote stone development. In the present study, urine specific gravity was a significant predictor of TSC-associated urolithiasis. We recommend to TSC patients and their families to drink > 2 L of water a day.

Medical expulsive therapy is recommended for urinary stones < 10 mm in size [16]. Alpha blockers promote the spontaneous expulsion of urinary stones [17]. On the other hand, urinary stones ≥ 10 mm in size are not expelled spontaneously. Thus, surgical treatments such as TUL, ESWL, and percutaneous nephrolithotomy (PNL) are recommended [18]. AMLs are often comorbid with TSC [19, 20]. Unlike sporadic AML, TSC-associated AML develops at multiple sites on the bilateral sides [7]. It develops at a younger age, and tends to exhibit a much faster growth rate over time than sporadic AML [21, 22]. When urolithiasis is comorbid with TSC-associated AML, ESWL, and PNL are difficult because of the risk of bleeding from AML. On the other hand, TUL can be performed safely. Surgical options for the treatment of urolithiasis in patients with TSC-associated AML are limited. Furthermore, topiramate and zonisamide lead to the development calcium phosphate stones in some cases. In the present study, only one stone was composed of pure calcium oxalate; 10 were composed of mixed calcium phosphate and calcium oxalate. Urinary stones containing calcium phosphate are harder than calcium oxalate stones [23]. Otsuki et al. reported that stones containing calcium phosphate require more laser energy and a longer operating time, and that they are associated with a higher rate of perioperative complications [23].

The present study has several limitations. First, this was a retrospective study and the urolithiasis group consisted of only 20 patients. The incidence of urolithiasis associated with TSC in this study does not reflect the overall prevalence of TSC in Japan. In addition, our hospital manages a large number of patients with refractory epileptic seizures; thus, the treatment period of patients receiving antiepileptic agents may be longer than in other medical institutions. Second, the urinary calcium and citrate concentrations were not measured in this study. The urine calcium to creatinine ratio and urine citrate to creatinine ratio change with diet and exercise. Spot urine examinations during early morning fasting, and 24-h urine collection are difficult in TSC patients with mental retardation. Further investigations are necessary to achieve a better understanding of the development of TSC-associated urolithiasis.

## Conclusions

Patients with TSC who have an increased urine specific gravity, alkaline urine, and a longer administration of topiramate and zonisamide tend to demonstrate an increased risk of developing urolithiasis and therefore such cases require adequate care. Periodic blood, urine, imaging tests and appropriate use of antiepileptic drugs are necessary. If urolithiasis is comorbid with TSC-associated AML, the treatment options are more limited in cases with multiple AMLs around the stone due to an increased risk of hemorrhage.

## Data Availability

Data are available on reasonable request.
